# Development and characterization of magnetic polyethyleneimine-bamboo nanocellulose adsorbents for enhanced performance of dye pollutant removal

**DOI:** 10.1186/s40643-025-00928-y

**Published:** 2025-08-08

**Authors:** Jameelah Alhadi Salih Othman, Norzita Ngadi, R. A. Ilyas, Abu Hassan Nordin, M. F. M. Alkbir, Mohd Nor Faiz Norrrahim, Victor Feizal Knight, Khalid A. Alzahrani, Anish Khan, Pijush K. Mondal

**Affiliations:** 1 Faculty of Chemical and Energy Engineering, University Technology Malaysia (UTM), Skudai, Johor 81310 Malaysia; 2 Faculty of Science and Arts, University Zintan, Badr, Libya; 3 Centre for Advanced Composite Materials (CACM), University Technology Malaysia (UTM), Skudai, Johor 81310 Malaysia; 4 Institute of Tropical Forestry and Forest Products (INTROP), University Putra Malaysia, Serdang, Selangor 43400 Malaysia; 5 Centre of Excellence for Biomass Utilization, University Malaysia Perlis, Arau, Perlis 02600 Malaysia; 6 Faculty of Applied Sciences, Universiti Teknologi MARA (UiTM), Arau, Perlis 02600 Malaysia; 7 Advanced Facilities Engineering Technology Research Cluster, Malaysian Institute of Industrial Technology (MITEC), University Kuala Lumpur, Johor Bahru, Malaysia; 8 Plant Engineering Technology (PETech), UniKL Malaysian Institute of Industrial Technology (MITEC), Persiaran Sinaran Ilmu, Johor Darul Takzim, Johor Bahru, Malaysia; 9 Research Centre for Chemical Defence, Defence Research Institute (DRI), Universiti Pertahanan Nasional Malaysia, Lumpur, Malaysia; 10 Chemistry Department, Faculty of Science, King Abdulaziz University, Jeddah, Saudi Arabia; 11 Center of Excellence for Advanced Materials Research, Faculty of Science, King Abdulaziz University, Jeddah, Saudi Arabia; 12 School of Advance Science & Languages, VIT Bhopal University, Bhopal-Indore Highway, Kothrikalam, Sehore, Madhya Pradesh 466114 India

## Abstract

The adsorption of reactive black 5 (RB5) onto bamboo nanocellulose modified with polyethyleneimine (PEI) and magnetic nanoparticles (MNPs), referred to as MNCPEI (Fe_3_O_4_, ~ 10–20 nm), were incorporated via direct dispersion during the synthesis process without surface pre-functionalization was systematically examined in aqueous solution. The efficiency of dye removal increased proportionally with the amount of MNCPEI in the modified adsorbents. Characterization studies revealed that the MNCPEI modification significantly enhanced the surface chemistry of the adsorbents while providing slight improvements in their textural properties. High removal efficiencies for the anionic dyes were consistently achieved, demonstrating the superior performance of the MNCPEI adsorbents. Optimal adsorption conditions were observed at a nanocellulose-to-PEI ratio of 1:2 and an MNP dosage of 0.3 g. The findings indicated that the adsorption of RB5 was governed by monolayer adsorption, with chemical adsorption identified as the rate-limiting step. The adsorbent exhibited a high maximum adsorption capacity (99%) for RB5, attributed to the increased availability of functional groups facilitating interaction with the dye molecules. Key parameters affecting the adsorption process, including contact time (0–180 min), adsorbent dosage (0.05–2.0 g), initial dye concentration (0.025–0.3 g/L), pH (2–9), and temperature (25–70 °C), were investigated. This study underscores the potential of bamboo-derived MNCPEI adsorbents for effective textile wastewater treatment.

**Graphic Abstract:**

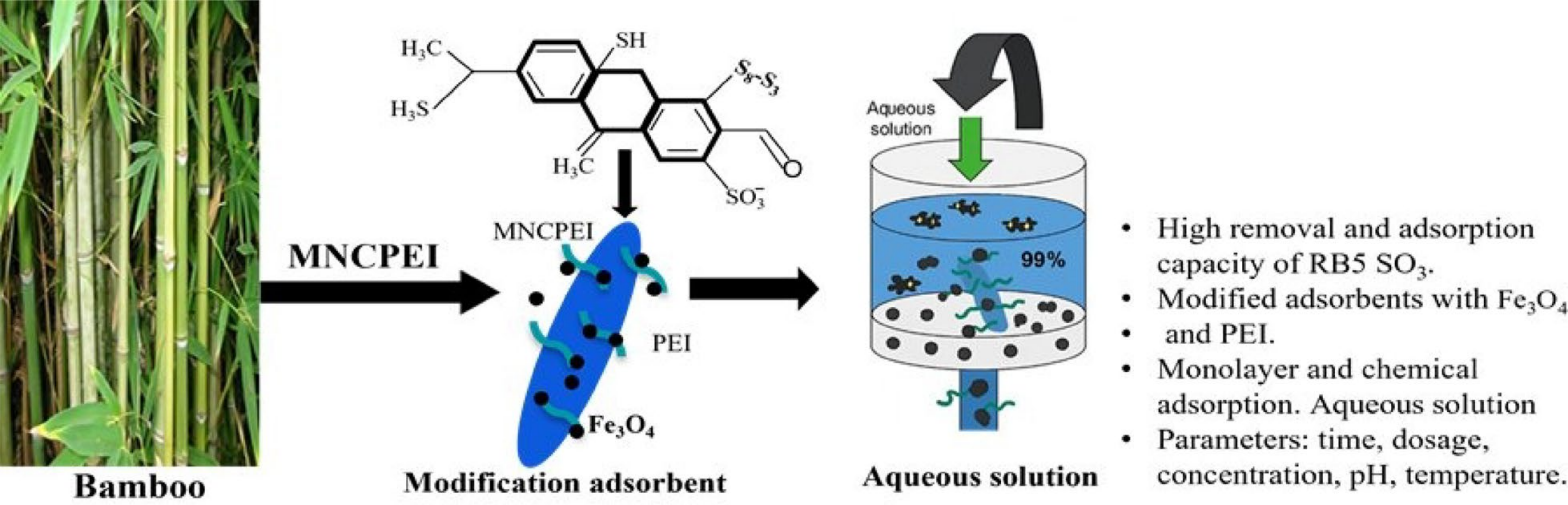

**Supplementary Information:**

The online version contains supplementary material available at 10.1186/s40643-025-00928-y.

## Introduction

The expansion of dye-based operations, such the textile industry, along with the accompanying increases in industrialization and urbanization, has recently made dye pollutants in aquatic resources a major concern. One of the most dynamic and rapidly expanding markets globally right now is the textile industry. The wastewater they generate is substantial and largely consists of dyestuff colors, which is a result of their high-water consumption (Panhwar et al. 2024). They are quite water-intensive and generate a tremendous amount of effluent, the majority of which is colored water from the dyeing process. Their effects on people’s daily lives are profound(Al-Tohamy et al. 2022a; Bukola et al. 2022). Dye pollution in aquatic environments can inhibit photosynthesis and impede the growth of aquatic flora and microorganisms by absorbing light and obstructing its penetration (Sharma et al. 2021). Reactive Black 5 (RB5) is commonly used as a representative anionic dye in wastewater treatment research due to its pronounced toxicity and strong resistance to degradation. Its environmental persistence is highlighted by a half-maximal effective concentration (EC50) of 27.5 mg/L, underscoring the challenges it presents in conventional remediation processes (Dutta et al. 2024). This suggests that even minimal releases of RB5 dye could negatively impact the environment and organisms.

Nanomaterials are increasingly employed for dye removal owing to their exceptionally high surface area and abundance of active sites, which facilitate more efficient interactions with dye pollutants. Nanomaterials can be sourced from a wide array of origins, encompassing both hardwood and softwood species, natural fibers such as flax, hemp, jute, ramie, and sandpaper-like plant fibers. Additionally, grasses like bagasse and bamboo serve as valuable feedstocks. Beyond terrestrial sources, nanomaterials can also be derived from biological entities including microorganisms, fungi, algae, and marine organisms, offering a rich and sustainable reservoir for nanomaterial production (Ahmed et al. 2024; Salama et al., 2021). Nanocellulose is an exceptional material for industrial water treatment systems due to its intrinsic qualities that render it suitable for wastewater treatment (Salama et al., 2021). Various research fields are showing growing interest in nanocellulose, which is a type of lignocellulosic-based material, because of its outstanding characteristics, including high aspect ratio, large specific surface area, excellent biocompatibility, and good chemical reactivity. The strong adsorption performance of nanocellulose is mainly attributed to its extensive surface area, which provides more available active sites for interaction with target substances.

Composites have been successfully produced by combining cellulose nanomaterials (CNMs) or cellulose nanofiber matrix with polyethyleneimine (PEI). This combination has attracted wide attention and has been applied in many industrial sectors, especially in the field of paper manufacturing, due to the improved functional and mechanical properties provided by the composite structure (Mo et al. 2022; Nan et al. 2023; Xie et al. 2020; Zheng et al. 2022), wastewater treatment (Hu et al. 2011; Wang et al. 2020), drug release (Hlongwane et al. 2019; Strokal et al. 2019), sensing (Guo et al. 2018; Liu et al. 2017), heterogeneous catalysis, and others (Liu et al. 2017). These composites have garnered significant interest due to their prospective applications, and the volume of papers in this domain has consistently risen over the past twenty years. It is significant that many of these studies (58.3%) concentrated on adsorption devices for water purification (Liu et al. 2017). MNPs have garnered considerable attention in recent years owing to their facile production, customizable surfaces, magnetic properties, and biocompatibility. Furthermore, its adaptability permits utilization across various applications. An extensive examination of magnetic nanoparticles demonstrates their significant promise in multiple domains (Katz 2019).

Nanocellulose is a naturally derived material that is widely used in the preparation of magnetic nanomaterials. In most cases, magnetic nanocellulose-based materials are fabricated by incorporating iron oxide particles into the nanocellulose matrix. This approach allows the resulting material to combine the unique properties of nanocellulose with magnetic functionality, making it suitable for various advanced applications (Wu et al. 2011). Magnetic nanocomposites (MNCs) have shown great potential in a variety of applications, including their use as recyclable catalysts and in the medical field (Wu et al. 2011; Xia et al. 2017). Additionally, nanocellulose combined with MNPs has been applied as an oil adsorbent (Daneshfozoun et al. 2017). In recent years, researchers have explored various advanced materials for water purification. For example, (Liu et al. 2025) developed a piezoelectric photocatalyst using Bi_2_S_3_-coated BaTiO_3_ nanorods that can degrade dyes under visible light, while(T. Wang et al. 2024a, b) created biomass aerogels with aligned channels designed for oil–water separation and solar-driven evaporation. Although these systems show promising results, they often involve complex synthesis steps, depending on external energy sources, or target different types of pollutants altogether. In contrast, our approach focuses on a simpler, low-energy method by modifying bamboo nanocellulose with polyethyleneimine and magnetic nanoparticles. Magnetic hybrid materials have been prepared by combining cellulose nanocrystals (CNCs) with cobalt-iron oxide particles, as reported by Jodeh et al. (2018). In addition, iron-based nanoparticles have shown good performance in removing various pollutants such as organic dyes and heavy metals from wastewater systems (Nizamuddin et al. 2019). The high adsorption efficiency of these nanoparticles is mainly related to their large surface area, strong interfacial activity, and magnetic properties, which make them suitable as effective adsorbents for toxic substances in contaminated water.

Although nanocellulose-based materials and polymer-modified adsorbents have been widely studied for dye removal, there are still areas that warrant further exploration. Many existing studies focus on removal efficiency alone, often overlooking optimization of material synthesis or long-term usability. Certain works have investigated how performance declines over repeated use or how synthesis variables influence both structural properties and functional performance. These gaps are important, especially when considering practical applications for wastewater treatment. Addressing these issues, the present work focuses on designing a bamboo-based nanocellulose adsorbent modified with polyethyleneimine and magnetic nanoparticles.

While MNCPEI demonstrated strong initial adsorption performance and reusability, its gradual efficiency loss over multiple cycles reflects a common challenge in biosorbents. Literature identifies common fatigue mechanisms including active site blockage, functional group deterioration, and structural destabilization over time (Elwakeel et al. 2025; Khanday et al. 2025). However, compared to other adsorbents such as PEI@Zein microparticles (Zhuang et al. 2023) or chitin-magnetite hybrids (Indira et al. 2023), MNCPEI maintains adsorption stability across a broad pH range (2–9), allows magnetic recovery in seconds, and avoids elaborate regeneration protocols like microwave desorption. These features position MNCPEI as a practical, cost-effective candidate for industrial dye removal especially where sustainable material sources and ease of reuse are essential.

Although RB5 is often considered resistant to degradation due to its stable azo and sulfonate groups, studies have shown that partial biodegradation is possible under specific enzymatic or microbial conditions (Benny and Chakraborty 2025; Rajendran et al. 2024). However, these methods often demand strict biological environments, longer treatment times, or specialized catalysts. In contrast, we adopted an adsorption approach for its ease of use, reusability, and quick response especially in settings where biodegradation is not practical. While RB5 was not chemically degraded here, our material effectively captured it through functional group interactions, offering a cleaner, low-energy solution rooted in renewable materials. The main objective of this study is to determine the best synthesis conditions for preparing a modified magnetic polyethyleneimine-bamboo nanocellulose (MNCPEI) adsorbent. The research emphasizes careful optimization of synthesis parameters to enhance the MNCPEI’s efficiency in removing Reactive Black 5 (RB5) dye from aqueous solutions. Detailed characterization was carried out to fully understand the physicochemical properties, magnetic behavior, and morphological features of the developed adsorbent. Special attention was given to examining the structural features and surface chemistry of MNCPEI. This work aims to produce a stable and efficient adsorbent that can be applied in water purification systems. The findings from this study are expected to contribute valuable knowledge to the field of environmental nanotechnology, especially in promoting the development of innovative adsorbents derived from renewable and eco-friendly materials.

## Methodology

### Preparation bamboo nanocellulose (BNFC)

Wet disc milling (WDM), produced by Grow Engineering in Adachi-ku, Tokyo, Japan, has two ceramic nonporous disc grinders with a configurable clearance of 20–40 μm between the upper and lower discs, functioning at a speed of 1800 rpm. Using the WDM technique, bamboo nano-fibrillated cellulose (BNFC) was produced from BC. Approximately 2 L of fibre suspension with a 2 wt% concentration were subjected to 20 cycles in the grinder. The resultant fibres were meticulously sealed and preserved at 2 °C for subsequent utilization. A 10 mL slurry sample was subjected to freeze-drying for subsequent analysis.

### Adsorbent synthesis and batch adsorption study

The MNCPEI adsorbent was produced via a modified cross-linking technique based on the research of (Sajab et al. 2013). During this procedure, nanocellulose, PEI, and MNPs were amalgamated in a water bath at 65 °C. Nanocellulose and PEI were mixed in different amounts (1:0.5, 1:1, and 1:2) to get different mixtures. The synthesis involved first dispersing bamboo-derived nanocellulose in distilled water, followed by the gradual addition of PEI in varying mass ratios (1:0.5, 1:1, 1:2) under continuous stirring at 65 °C. MNPs were then introduced in controlled dosages (0.3–2.5 g), allowing uniform integration through a water bath-assisted mixing process. Crosslinking was initiated by adding glutaraldehyde (1–10% v/v), and the mixture was stirred and incubated for 0.5 to 24 h, depending on the formulation. This approach is adapted and modified from (Sajab et al. 2013), with improvements to enhance magnetic response and surface reactivity for dye adsorption. Similar strategies have been reported in literature for PEI-modified nanocellulose composites in dye removal (S. Ahmed et al., 2017; Sun et al., 2022a).

**Fig. 1:**
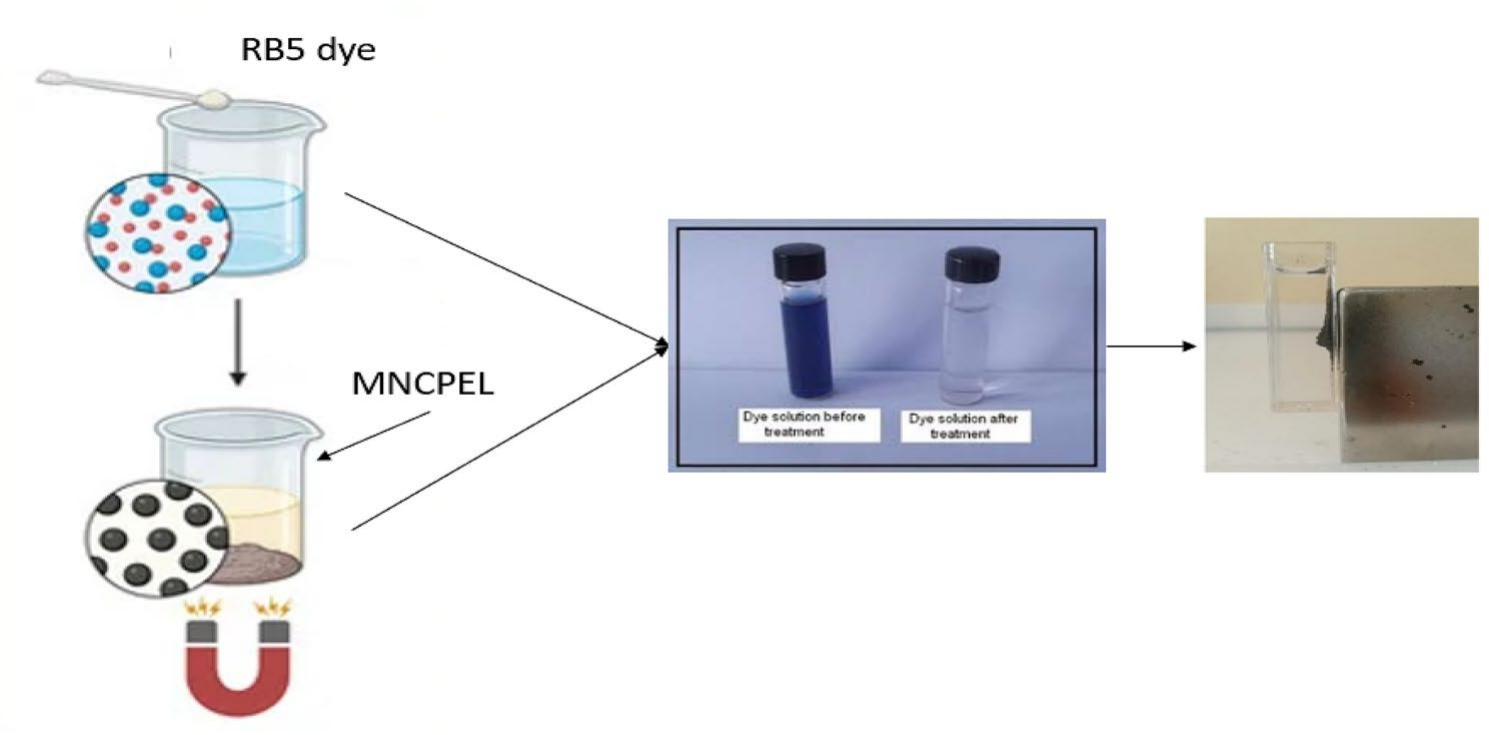
Schematic illustration of adsorption and separation of RB5 from synthetic dye

Batch adsorption was performed in 50 mL sample vials, each containing 0.1 g of the synthesized adsorbent along with a 0.1 g/L RB5 dye solution. The vials underwent agitation at 200 rpm for a duration of 60 min, maintained at ambient temperature. Following the reaction, the adsorbent was removed from the solution (see Fig. 1), and the absorbance of the filtrate was measured. The RB5 dye solution, created by dissolving RB5 powder in distilled water, was examined with a UV-vis spectrometer (Shimadzu UV-1280, Japan), revealing a distinctive peak at 598 nm. The objective of the adsorption experiments was to evaluate the impact of numerous factors on the cleanup of RB5 dye by employing the developed MNCPEI adsorbent. Conditions included contact time (0–180 min), starting RB5 dye content (0.025–0.3 g/L), temperature (25–70 °C), adsorbent dose (0.05–2.0 g), and pH (2–9). A standard calibration curve at this wavelength was used to find the starting and final concentrations of RB5. Using Eqs. (1) and (2), the concentration numbers were used to find the percentage of RB5 removal alongside the adsorbent capacity. The resultant data were subsequently utilized for kinetic, isothermal, and thermodynamic modelling.

1 $${\rm{Percentage }}\,{\rm{removal }}\left({\rm{\% }} \right){\rm{ = }}{{\left({{{\rm{C}}_{\rm{0}}}{\rm{ - }}{{\rm{C}}_{\rm{F}}}} \right)} \over {{{\rm{C}}_{\rm{0}}}}}\,{\rm{ \times }}\,{\rm{100}}$$

2 $${\rm{Adsorption}}\,{\rm{ capacity }}\left({{\rm{qe}}} \right){\rm{ = }}{{\left({{{\rm{C}}_{\rm{0}}}{\rm{ - }}{{\rm{C}}_{\rm{f}}}} \right)\, \times \,V} \over {\rm{m}}}$$

where Co and Cf (mg/L) are the initial and the final concentrations of RB5, respectively, V (L) is solution volume and m (g) is adsorbent dosage.

The choice of a 1:2 nanocellulose-to-PEI ratio was made after preliminary testing showed it offered the best balance between adsorption efficiency and material consistency. Although the adsorption differences between ratios may not appear large, this formulation provided more stable coating on the nanocellulose and a higher density of amine groups, both of which supported better dye interaction, especially under varied pH conditions. Lower ratios like 1:0.5 or 1:1 occasionally led to weaker performance or less uniform dispersion of magnetic nanoparticles. Based on these observations, the 1:2 ratio was selected as the most effective and reliable for the rest of the study.

### Characterization

The surface chemical characteristics of nanocellulose and the modified magnetic bamboo nanocellulose polyethyleneimine (MNCPEI) adsorbent were analyzed using a Fourier Transform Infrared (FTIR) spectrophotometer (IRTracer-100, Shimadzu, Japan). The scanning was performed in the range of 500 to 4000 cm⁻¹ with a resolution of 2 cm⁻¹. The specific surface area, pore diameter, and pore volume of the adsorbents were determined using nitrogen adsorption-desorption isotherms in combination with the multipoint Brunauer–Emmett–Teller (BET) method. Magnetic properties, including hysteresis behavior, were investigated using a vibrating sample magnetometer (VSM, Lakeshore Model 17,404) for both unmodified magnetic nanoparticles (MNPs) and MNCPEI samples. Furthermore, the point of zero charge (pHₚzc) of MNCPEI was evaluated by applying the solid addition technique. In this method, 50 mL of 0.1 mol/L KNO₃ solution was placed in a series of 100 mL bottles, each adjusted to different initial pH values ranging from 2 to 10 using 0.1 M HCl or NaOH solutions. This analysis helps to understand the surface charge behavior of the adsorbent under varying pH conditions.

Afterward, each bottle was treated with 0.1 g of MNCPEI, and the mixture was agitated at 30 rpm for 24 h. A filter was employed to extract the mixture, and the ultimate pH of the solution was determined (Gonçalves et al. 2024). The content of amine groups in the samples was measured through acid-base titration, as described by (Ge et al. 2016; Nordin et al. 2021). In simple terms, 10 mL of 0.1 M HCl solution was used to immerse 0.05 g of nanocellulose-PEI samples, which were then agitated at 150 rpm for 8 h. Following this, 2.5 mL of the supernatant was extracted and diluted to 25 mL with distilled water. After adding two droplets of phenolphthalein as an indicator, the mixture was titrated with a 0.01 M NaOH solution until a colour change was noticed. Following the advice of, we used Eq. (3) to determine the concentration of amine groups in the samples (Ge et al. 2016).

3 $$\eqalign{ & {\rm{Amine}}\,{\rm{group}}\,{\rm{contents}}\,\left({{\rm{mol }}{{\rm{g}}^{{\rm{ - 1}}}}} \right) \cr & {\rm{ = }}{{{C_1}{V_1} - {C_2}{V_2}} \over m} \times 16 \cr} $$

where, C₁ and C₂ represent the molar concentrations (M) of hydrochloric acid (HCl) and sodium hydroxide (NaOH), respectively. V₁ and V₂ are the volumes (L) of the HCl and NaOH solutions used during the titration process. m refers to the mass (g) of the adsorbent sample.

### Reusability studies

The reusability of an adsorbent is an important factor to be considered when selecting a suitable and cost-effective sorbent for practical or pilot-scale adsorption applications. To evaluate the reusability performance, an adsorption experiment was conducted using 0.1 g of the magnetic polyethyleneimine-modified nanocellulose (MNCPEI) adsorbent, which was introduced into 50 mL of a 0.1 g/L dye solution adjusted to pH 7. The mixture was agitated at 200 rpm for 180 min at a controlled temperature of 27 °C to ensure equilibrium was reached.

After adsorption, the dye-loaded adsorbent (0.1 g) was separated and subjected to a desorption treatment using 100 mL of hydrochloric acid (HCl) and sodium hydroxide (NaOH) solutions with different concentrations (0.1 M, 1 M, and 5 M). The desorption step was also carried out at 27 °C for 30 min under the same stirring speed of 200 rpm. The recovered adsorbent was thoroughly rinsed with distilled water until neutral pH was achieved, followed by drying in an oven until a constant weight was obtained. The regenerated adsorbent was then reused in a new adsorption cycle to assess its efficiency. This adsorption-desorption process was repeated for four consecutive cycles to investigate the material’s regeneration potential and stability during repeated use.

## Results and discussion

### Adsorbent characterization

#### Analysis of functional group

**Fig. 2:**
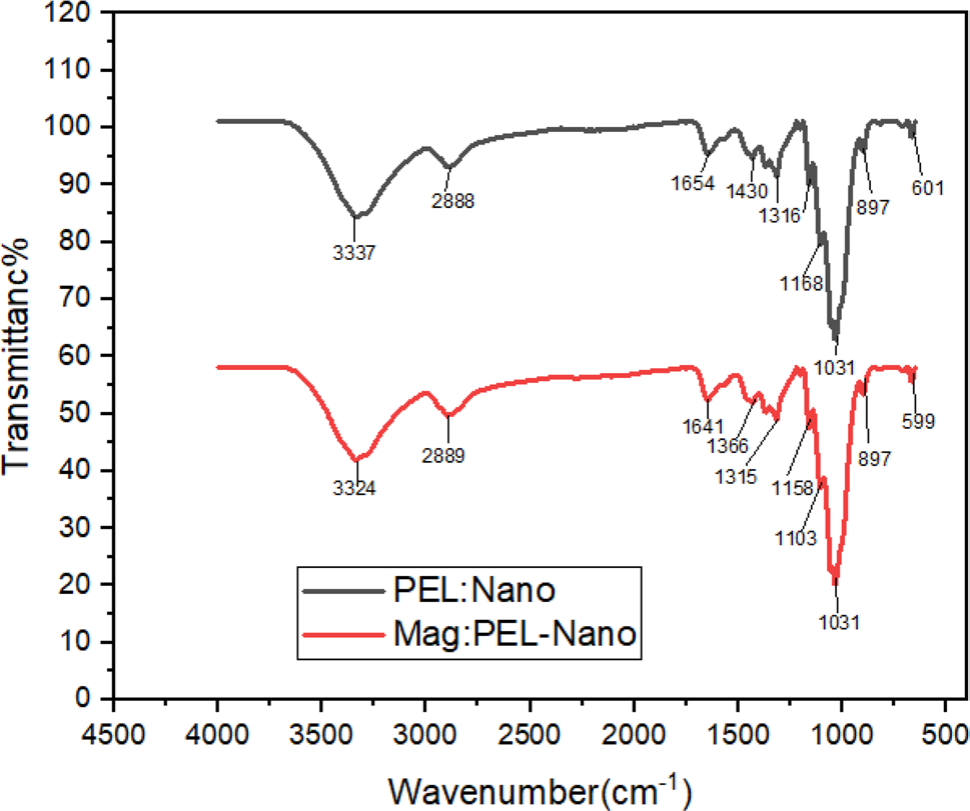
FTIR spectra of PEI-nanocellulose and MNCPEI adsorbents

NC-PEI and MNCPEI’s FT-IR transmittance spectra are illustrated in Fig. 2. BNC-PEI’s absorption peak at 1654 cm⁻¹ is a result of the stretching vibration of the C = N group, which is generated during the cross-linking of the hydroxyl group of nanocellulose and the amine group of PEI. This demonstrates the successful cross-linking of PEI with BNFC (Guo et al. 2024). Several significant absorption peaks characteristic of cellulose have also been identified in the FTIR spectrum. The peak observed at 1160 cm⁻¹ corresponds to the C–O–C stretching vibration of the β-glycosidic linkage. The band at 1316 cm⁻¹ is attributed to the vibration of the–COOH functional group, while the peak at 1430 cm⁻¹ is related either to–CH₂ bending or the symmetric stretching of the–COO⁻ group, commonly found in its salt form. Additionally, the peak near 2886 cm⁻¹ indicates aliphatic–CH stretching vibrations, and the broad absorption at 3337 cm⁻¹ is due to the–OH stretching vibrations, reflecting the hydroxyl groups present in the cellulose structure (Kim et al. 2018). These peaks confirm the presence of typical cellulose functionalities, which play a key role in chemical interactions and adsorption behavior.

A strong adsorption peak at 599 cm^− 1^ stands out when compared to the MNCPEI spectra. The effective synthesis and attachment of nano Fe_3_O_4_ to the MNC surface was demonstrated by this peak, which was associated with the Fe-O stretching vibrations in Fe_3_O_4_. One identifying feature of Fe-O in tetrahedral sites is a wideband with a peak intensity at around 599 cm⁻¹ (Lesiak et al. 2019). Typically, this band is observed at around 600 cm^− 1^ or slightly below, according to the literature (Helmiyati and Anggraini 2019). The transition of this band to a reduced wave number results from the establishment of Fe–O bonds on the nanocellulose surface. The diminished strength of this signal results from the decreased concentration of Fe_3_O_4_. The displacement of the O–H peak following the deposition of Fe_3_O_4_ on the NC surface signifies an interaction between cellulose and the magnetic nanoparticles (Janićijević et al. 2022). Absorption peak at 1336 cm^− 1^ corresponds to the stretching vibration of C–N as a result to the grafting of PEI (Li et al. 2018).

The observed functional groups also play a central role in the adsorption mechanism of RB5 onto MNCPEI. The presence of abundant primary and secondary amines from PEI introduces positive surface charges under acidic to neutral pH conditions, promoting electrostatic attraction with the negatively charged sulfonic acid groups in RB5 (Al-Araji et al. 2023; Chen et al. 2020). Additionally, the hydroxyl groups from nanocellulose contribute to hydrogen bonding with the azo and sulfonic moieties of the dye. These interactions are likely responsible for the strong affinity between MNCPEI and RB5 (Liyanapathiranage et al. 2020). Furthermore, although less dominant, π–π interactions may occur between the aromatic rings of the dye and any residual unsaturated regions within the polymer matrix(Gospodinova and Tomšík 2015). These combined mechanisms support the high adsorption efficiency observed and align with trends reported in the literature for polyamine-functionalized biosorbents (Mahadevi and Sastry 2013). This mechanistic insight, supported by FTIR spectra and adsorption behavior, reinforces the role of functional group availability and surface chemistry in dye removal performance (Badsha et al. 2021; Fan et al. 2018).

Recent studies have continued to emphasize the importance of functional group availability—particularly amines and hydroxyls—in promoting strong electrostatic and hydrogen bonding interactions with dye molecules. For example, (Yuan et al., 2024) demonstrated enhanced anionic dye removal using PEI-functionalized cellulose with similar surface interactions. Likewise, (Baraka & Labidi, 2024) noted that amino-rich biosorbents facilitate rapid dye adsorption through both surface complexation and ion-exchange mechanisms. In the case of sulfonic acid-containing azo dyes like RB5, these interactions are especially pronounced at neutral to acidic pH levels due to increased protonation of amine groups. These findings align with our results, where the presence of PEI contributed significantly to high RB5 removal efficiency. Moreover, recent work by (Hashem et al., 2024) and(Pormazar and Dalvand 2022b) support the notion that modified nanocellulose can act as a multifunctional adsorbent, combining high surface area with specific chemical reactivity to enhance pollutant capture.

#### Surface area and pore analysis

The adsorption capacity of porous materials is significantly influenced by their surface area and porosity, which are essential characteristics for their application as adsorbents (Paredes-Quevedo et al. 2021). Table 1 presents the results for the surface area, average volume, and pore size distribution of the adsorbents. Nanocellulose possesses a surface area of 6.25 m²/g, highlighting its potential as a material with a substantial interface for improved performance. Cellulose nanofibers derived from wheat straw exhibited a substantial surface area of 6–15 m²/g (Chen et al. 2010). Additionally, (George & S N, 2015) reported a high 5m^2^/g surface area of nanocrystals CNCs from cotton. This feature offers several advantages, including improved adhesion, enhanced surface functionalization, and better dispersion quality in polymer matrices.

The magnetic nanocellulose-PEI and nanocellulose-PEI had BET surface areas of 2.65m^2^/g and 5.03m^2^/g, respectively, with pore volumes of 0.0146 cm^3^/g and 0.0075 cm^3^/g. The change decreased the surface area of nanocellulose from 6.25 cm^3^ to 5.03 cm^3^ (nanocellulose-PEI). A prior study found that adding PEI to the adsorbent sodium silicate reduced its surface area from 994 m²/g to 291 m²/g. The pore interior was partially filled with PEI, preventing N₂ gas molecules from entering, resulting in a decreased surface area and pore volume (Ahmed et al., 2017). The reduction in average pore size following modification resulted from the integration of amine groups into the pore architecture. Nonetheless, this reduction did not influence the adsorption rate of RB5, and the surface area of MNCPEI diminished to 2.65 m^2^/g (Liu et al. 2021).

**Table 1:** Textural properties of adsorbents

Adsorbent	Surface area (m^2^/g)	Pore volume (cm^3^/g)	Pore size (nm)
Nanocellulose	6.25	0.0074	10.278
PEI-Nanocellulose bamboo	5.03	0.0075	6.0343
Magnetic-PEI-Nanocellulose	2.65	0.0146	2.2103

The magnetic nanocellulose-PEI composite’s BET surface area decreased from 5.03 to 2.65 m²/g due to magnetite aggregation, thus limiting the exposed surface area (Kondor et al. 2021). The increased pore volume (from 0.0075 to 0.0146 cm³/g) suggests that while larger pores are introduced, smaller pores that contribute more to surface area are blocked or filled. The surface area of the PEI-modified nanocellulose decreased from 8.5 m^2^/g to 4.2 m^2^/g when magnetite was precipitated onto it, compared to the surface area of the polyethylene amine-modified nanocellulose without magnetite, which had been previously added to it through a chemical reaction process (Tamo 2024).

#### Magnetic properties

**Fig. 3:**
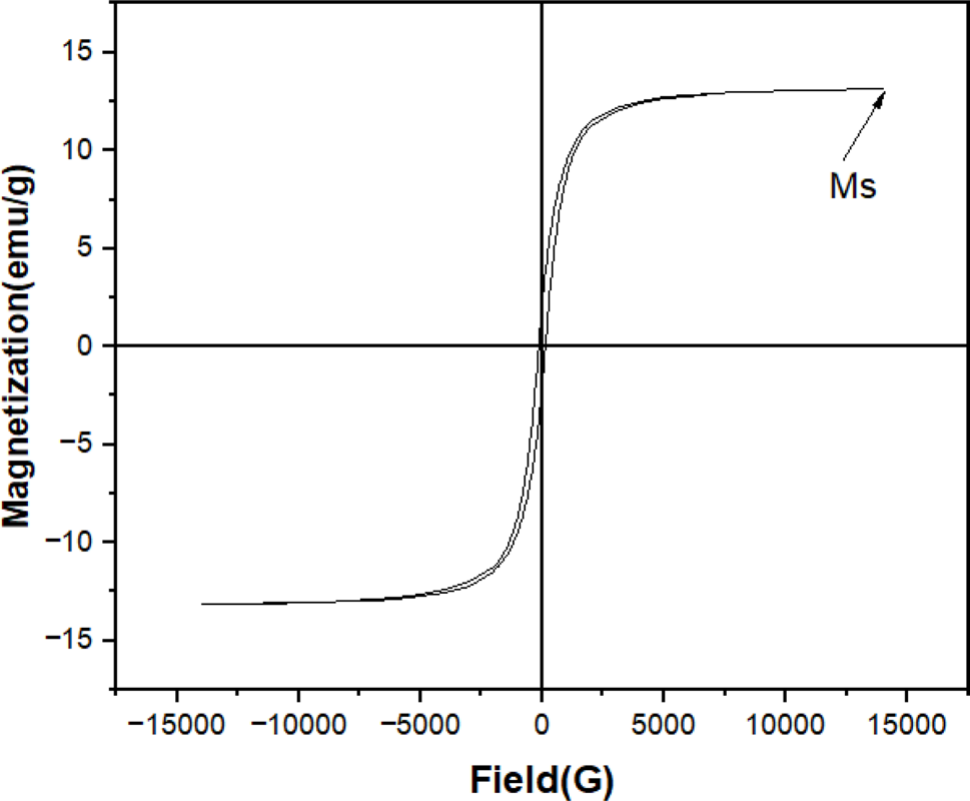
Magnetization of MNCPEI

The magnetic properties of NMCPEI were investigated via hysteresis curves provided by VSM. The magnetization curve of MNCPEI adsorbent (Fig. 3) indicates the MNCPEI (before and after adsorption) was 13.40 and 13.43 emu/g, respectively. The material’s strong magnetic properties are beneficial for magnetic adsorbents. This allows simple separation of the material from a solution using an external magnetic field after adsorption.

The lower Ms value of the MNCPEI adsorbent compared to bare MNP is due to the nonmagnetic response of the silane coupling agent and the polymer matrix. However, MNCPEI still retains a certain magnetic response (Harvell-Smith et al. 2022). In addition, it was apparent that all magnetic materials are superparamagnetic due to their zero remanence and coercivity (Wallyn et al. 2019). Also, the fact that the Ms value is lower after PEI production indicates that the magnetic nanoparticles and non-magnetic nanocellulose are more strongly attracted to one other by the magnetic field, which keeps them from becoming detached (Vohl et al. 2024). Additionally, the reduced Ms value suggests an improved magnetic field attraction between the magnetic nanoparticles and the non-magnetic nanocellulose with PEI, preventing the detachment of these materials after synthesis (Sharma et al. 2018; Zhang 2016). Within ten seconds of adsorption, the adsorbent can be readily isolated and made available for reuse. In this regard, future magnetic separation and recovery of MNCPEI from wastewater containing dyes will depend on properly calibrated magnetic responsivity.

### Effect of adsorbent preparation parameters

#### Effect of ratio between nanocellulose to PEI

The impact of a particular nanocellulose to PEI mass-to-volume ratio on RB5 elimination under stated conditions is shown in Fig. S1(a), which included a 4-hour impregnation time, a 30-minute crosslinking time, and a 1% (v/v) glutaraldehyde sample. Adsorption efficiencies and capacities of 90.70%, 91.75%, and 93.64%, respectively, were achieved by samples with nanocellulose-to-PEI ratios of 1:0.5, 1:1, and 1:2. Adsorption efficiency is improved by increasing the amount of PEI-modified nanocellulose, as shown above($ et al. 2022a). Amino groups are introduced to nanocellulose by PEI modification, which enhances its binding capability to heavy metals, dyes, and other contaminants. Adsorption efficiency requires immobilization of PEI onto a solid matrix that offers accessible active sites (Yuan et al., 2024). Table 2 shows that the nanocellulose: PEI ratios with the highest amine group concentrations are 1:2, 1:1, and 1:0.5, with values of 13.4, 13.6, and 13.5 correspondingly.

**Table 2:** The concentration of amine groups on adsorbents

Nanocellulose: PEI	Amine Group Content (mol g^− 1^)
1:0.5	13.4
1:1	13.6
1:2	13.5

These results are in line with the adsorption data, which showed that adsorbents with a higher nanocellulose content removed more RB5 than those with a lower nanocellulose content. This is because the nanocellulose surface has more active sites. Moreover, surface modification of nanocellulose not only enhances its functionality but also improves its organic adsorption properties (Salama et al., 2021; Si et al. 2022; Zoppe et al. 2020). Chemical alterations, such as the insertion of positively charged groups by cationic modifications with amino groups, are commonly used to make nanocellulose adsorbents for anionic dyes. Due to the uniform distribution of nanocellulose and PEI, the outcome is that their interaction is maximal; so, increasing the concentration of either component has no effect on the adsorption capacity, since all accessible sites may have already attained their maximum adsorption capacity (Yu et al. 2020).

#### Effect of impregnation time

When PEI solution interacts with solid-like nanocellulose, liquid-solid intermolecular forces come into play, causing PEI to concentrate and deposit on the nanocellulose surface (Baraka & Labidi, 2024). Under these circumstances, Fig. S1(b) shows how the RB5 elimination percentage and adsorption capacity are affected by the impregnation time: 1% (v/v) glutaraldehyde, nanocellulose, and a PEI ratio of 1:2, crosslinked for 30 min. When the impregnation time was prolonged from 30 min to 4 h, the results showed that the RB5 removal and adsorption capacity went up from 30.89% (15 mg/g) to 93.82% (47 mg/g). However, as the impregnation periods were decreased, the results showed a decline. At a reduced impregnation time (30 min to 2 h), insufficient contact time between the amine groups of PEI and the hydroxyl groups of nanocellulose likely hinders complete adsorption of PEI onto the nanocellulose surface. According to (Zhang et al. 2022a, b), this could be attributed to the slower adsorption rate between PEI and nanocellulose, resulting in fewer amine groups being adsorbed on the nanocellulose surface within a short time. The creation of hydrogen bonds between nanocellulose and water molecules is likely responsible for the substantial interactions observed after an impregnation duration exceeding 4 h (Chami Khazraji and Robert 2013).

As reported by Chami Khazraji and Robert (2013), nanocellulose chains possess a strong affinity toward substances containing hydroxyl groups, such as water molecules. Due to this characteristic, water can easily interact with the nanocellulose surface. This interaction creates a barrier that hinders the adsorption of anionic Reactive Black 5 (RB5) molecules onto the MNCPEI surface, primarily due to electrostatic repulsion between the negatively charged RB5 dye and the hydroxyl-rich hydrated nanocellulose, which also carries partial negative charges. Although this repulsive interaction did not significantly affect the overall adsorption efficiency during the 4-hour impregnation process, it is believed to have played a role in slightly reducing the dye uptake capacity of the MNCPEI adsorbent.

#### Effect of crosslinking time

A nanocellulose to PEI ratio of 1:2, an impregnation time of 4 h, and a glutaraldehyde concentration of 1% (v/v) are shown in Fig. S1(c). The figure shows how the length of the GTA crosslinking affects the removal of the RB5 dye. The data show that the clearance rate of RB5 dye increased with increased crosslinking time, reaching 96.11% (48.05 mg/g) after 30 min. After 30 min, no significant change in dye clearance percentage was seen. This trend corresponds with another study on the adsorption of organic dyes by diverse adsorbents, including nanocellulose-PEI and chitosan modified with GTA, which indicated that extended crosslinking duration generally enhances the dye removal rate to a certain degree, beyond which additional crosslinking yields minimal improvements (Bassyouni et al. 2022). Research on magnetic-PEI-cellulose indicated that dye removal effectiveness improved with crosslinking for approximately 30 min.

After this ideal duration, further crosslinking resulted in minimal to no enhancement in adsorption capacity, either to the saturation of active binding sites or structural alterations in the adsorbent that restrict subsequent adsorption capability (Zhao et al. 2017). The minimal removal % noted for adsorbents produced with brief crosslinking durations (10–20 min) can be ascribed to inadequate crosslinking of the PEI amine groups to the nanocellulose backbone via GTA, hindering the establishment of a fully developed copolymer chain. The adsorbent possesses a low density of active sites, resulting in a reduced percentage of RB5 dye removal from the solution (Adegoke and Bello 2015). As a result, the best crosslinking length is 30 min, as increasing the time does not enhance the percentage removal.

#### Effect of glutaraldehyde concentration

The results obtained in Fig. S1(d) indicate that GTA concentration plays a critical role in RB5 dye removal, which proceeds via Schiff base-mediated bridging among nanocellulose, PEI, and polysaccharide networks. This effect was evaluated under the conditions; nanocellulose: PEI ratio of 1:2, 4-hour impregnation period, and 30-minute crosslinking time. The optimal concentration of GTA in this study was 11% v/v, with RB5 dye removed at 96.07% (48.03 mg/g). A reduction in GTA concentration to 0.5% v/v led to a decline in adsorption performance, with the removal efficiency dropping to 90.44%, corresponding to an adsorption capacity of 45.22 mg/g.

The diminished crosslinking density in the adsorbent likely accounts for the noted decrease in RB5 dye removal effectiveness at lower GTA concentrations, specifically 0.5% v/v. GTA acts as a crosslinking agent by forming covalent bonds between the amine groups on PEI or nanocellulose fibres, thereby enhancing the structural integrity of the adsorbent and increasing the availability of binding sites for dye molecules (Sun et al., 2022). Because fewer crosslinks are formed at lower GTA concentrations, the adsorbent’s structure becomes less stable and there are less available adsorption sites, which reduces the RB5 dye absorption capacity (Mengelizadeh and Pourzamani 2020). However, increasing the GTA concentration to 10% (v/v) reduced RB5 dye removal to 74.51% (37.25 mg/g). This phenomenon is likely due to the restricted diffusion of pollutants through the polymer network at higher crosslinking densities, as noted by (Crini et al. 2019). Consistent with earlier findings, the quantity of free amine groups that can interact with pollutants is an important factor in RB5 removal.

#### Effect of magnetic nanoparticle (MNP) dosage

Nanocellulose-PEI was incorporated with MNPs to enhance particle separation. Fig. S1(e) depicts the impact of MNP dosage on the RB5 dye binding affinity. The effect was examined under the following conditions: a nanocellulose: PEI ratio of 1:2, an impregnation period of 4 h, a crosslinking time of 30 min, and a glutaraldehyde concentration of 1% v/v. The results indicated that the incorporation of a maximal quantity of MNP (0.1 to 0.6 g) into the adsorbent had no substantial effect on the percentage of RB5 eliminated or adsorbed. The percentage elimination and adsorption capabilities of RB5 diminished from 83.83% (41.91 mg/g) to 82.12% (41.06 mg/g) as the MNP dosage rose from 1 to 2 g. This may result from elevated MNP concentrations obstructing or partially occupying adsorption sites on the nanocellulose-PEI network. The surplus MNP may occupy sites that would otherwise be available for dye binding, so marginally diminishing adsorption capacity instead of improving it (Sathasivam et al. 2023), and the nanocellulose-PEI will become easily detached from magnetic iron in solution.

**Fig. 4:**
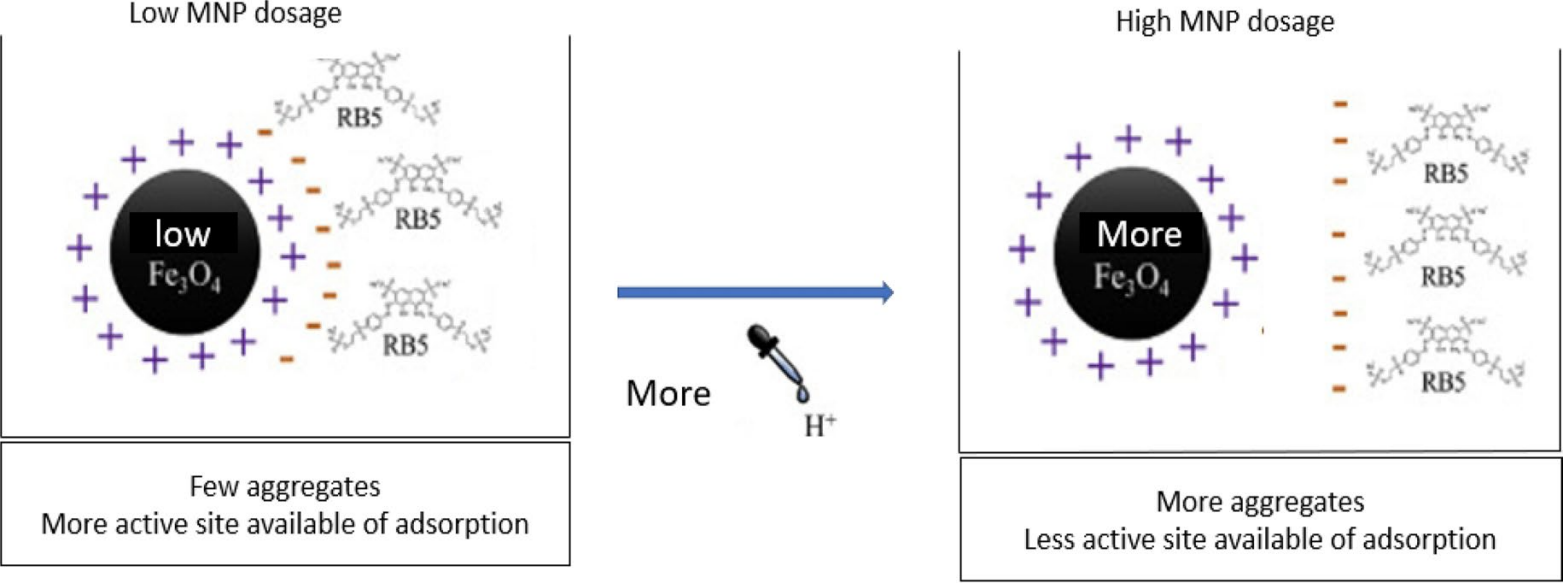
The effect of MNP dosage on the aggregation of adsorbent

Previous studies have indicated that the aggregation or clustering of nanoparticles can significantly reduce the number of accessible active sites for adsorption, thereby lowering the adsorption rate of RB5 molecules per unit mass of the adsorbent (Pormazar & Dalvand, 2022). This phenomenon leads to a reduction in the overall surface area and limits the availability of functional sites required for effective dye binding, as illustrated in Fig. 4. As a result, the adsorption performance decreases noticeably. Similar findings were reported in the adsorption behavior of methylene blue (MB), where an increase in adsorbent concentration led to a considerable decline in removal efficiency due to particle aggregation and site saturation (Birniwa et al. 2022). The findings were also consistent with Williams et al.‘s comparative research of magnetic and non-magnetic colloids (Williams et al. 2016). Consequently, the most effective quantity of MNP to combine with nanocellulose-PEI is 0.3 g, as this quantity guarantees the adsorbent’s uniform and homogeneous dispersion in the solution while preserving its exceptional adsorption capacity. The insights acquired during the synthesis of the adsorbent may prove advantageous in the advancement of cellulose-based biosorbents and other practical uses in wastewater treatment.

### Adsorption factors

#### Effect of contact time

Figure S2(a) presents the impact of varying reaction times on RB5 dye removal using MNCPEI, conducted at pH 7, 27 °C, with 0.1 g of adsorbent and an initial dye concentration of 0.1 g/L. The reaction durations investigated ranged between 5 and 240 min. Marginally extending the contact time enhanced the adsorption efficacy of RB5. The remarkably elevated clearance percentage, between 98% and 99.61%, validated the rapid RB5 adsorption rate. The functional groups on the adsorbent provided abundant active sites, greatly enhancing the adsorption efficiency for RB5 and attaining approximately 99% removal. A similar trend was reported in a study where PEI-treated coffee waste was employed for the adsorption of RB5 and CR dyes (Wong et al., 2020). The initial rapid dye uptake is due to the abundance of available binding sites on MNCPEI that quickly interact with RB5 molecules in the solution. As the adsorption continues, the availability of active sites becomes limited, causing a decline in the adsorption rate. The appearance of a plateau reflects the establishment of equilibrium, where dye molecules attach to and detach from the adsorbent surface at equal rates (Hashem et al., 2024a).

#### Initial dye concentration

The data illustrated in Fig. S2(b) suggest that initial RB5 concentration plays a key role in its adsorption onto MNCPEI, with concentrations between 0.05 and 0.3 g/L yielding a maximum removal rate of 99.61%. An increase in the starting dye concentration raises the overall amount of dye molecules within the fixed volume of solution and adsorbent mass. Higher concentrations lead to more dye molecules interacting with the active sites of the adsorbent, thereby improving the adsorption performance. This elucidates the rise in adsorption capabilities of MNCPEI from 26.30% at a concentration of 30–99.26% at a concentration of 5%. Moreover, elevated initial dye concentrations result in intensified competition for the finite active sites, culminating in the saturation of these sites and leaving a greater number of dye molecules scattered in the solution without adsorption (Wong et al., 2020). Therefore, it can be inferred that higher dye concentrations tend to lower adsorption efficiency. However, the adsorption capacity rises as the nanocomposite’s active sites become saturated under these increased concentrations (salah omer et al. 2022). The adsorption of methylene blue (Kavci et al. 2023) and brilliant green (Ali et al. 2017) applying dyes to peganum harmala-L seeds, canola straw, and calcium alginate all produced comparable results.

It is important to note that Reactive Black 5 (RB5), the target dye used in this study, is a complex anionic azo dye characterized by functional groups such as sulfonic acid moieties, azo bonds (-N = N-), and aromatic rings. While this work focused primarily on the adsorption efficiency of MNCPEI, these chemical groups are relevant to the dye’s interaction behavior. The amine-rich PEI-modified surface of the adsorbent likely interacts with the sulfonic and azo groups via electrostatic attraction and hydrogen bonding. However, the scope of the current study did not extend to the chemical degradation or cleavage of these functional groups. Therefore, RB5 is treated here as a non-degradable dye in the context of the applied material, with adsorption serving as the principal removal mechanism.

#### Effect of temperature

Another important aspect that affects the efficiency of adsorption is temperature. There was no discernible change in adsorption efficiency throughout a wide temperature range, as shown in Fig. S2 (c), which shows how temperature affects the elimination of RB5 dye. At 27 °C, the proportion of RB5 removed was around 99.61% and stayed the same up to 70 °C. The reason behind this behaviour is that when the temperature increases, the MNCPEI adsorbent’s pores and surface area grow larger. For the subsequent experiments, 27 °C will be used to minimize energy consumption. In a related study, (Ge et al. 2018) found that adsorption performance remained stable regardless of the temperature. Strong, temperature-independent adhesive forces between the adsorbent surface and the dye molecules prove the stability of the material. According to most researchers, the adsorbents developed in this study exhibit temperature-dependent behavior, including (Mpatani et al. 2020). Consequently, this uniformity of quality is very useful in water treatment procedures that take place at varying temperatures.

#### Effect of adsorbent dosage

The amount of adsorbent used is another crucial factor that influences its adsorption capacity during the process (Adegoke et al. 2023). Figure S2(d) illustrates how varying the adsorbent dosage influences RB5 dye removal efficiency at an initial concentration of 0.1 g/L, under conditions of 27 °C, pH 7, and a contact time of 120 min. An increase in adsorbent dosage from 0.05 g to 2.0 g led to a rise in RB5 dye removal efficiency, improving from 60.13 to 99%. Greater adsorbent dosages provide more surface sites to interact with dye molecules, leading to this improvement (Hoong and Ismail 2018). This pattern aligns with the earlier findings reported by Thorat et al., (2021) on RB5 dye removal using bacterial nanocellulose/polyethyleneimine (PEI/BC). Nevertheless, when the adsorbent dosage reached 2 g, a minor decline in dye removal was observed, dropping from 99.22 to 99.10%. This reduction in adsorption performance is most likely attributed to the saturation of active sites on the adsorbent surface, which limits the further attachment of dye molecules (Binaeian et al. 2020).

In addition, at higher adsorbent dosages, particle agglomeration may occur, resulting in a decrease in the effective surface area and thus a lower number of available adsorption sites (Obayomi et al. 2024). Despite this limitation, the removal efficiency of Reactive Black 5 (RB5) dye in this study consistently remained above 90% across all tested adsorbent dosages. This finding clearly indicates the high adsorption capability of the synthesized material and suggests that effective dye removal can still be achieved even at lower adsorbent dosages, highlighting its potential for economical and efficient wastewater treatment.

#### Effect of pH

**Fig. 5:**
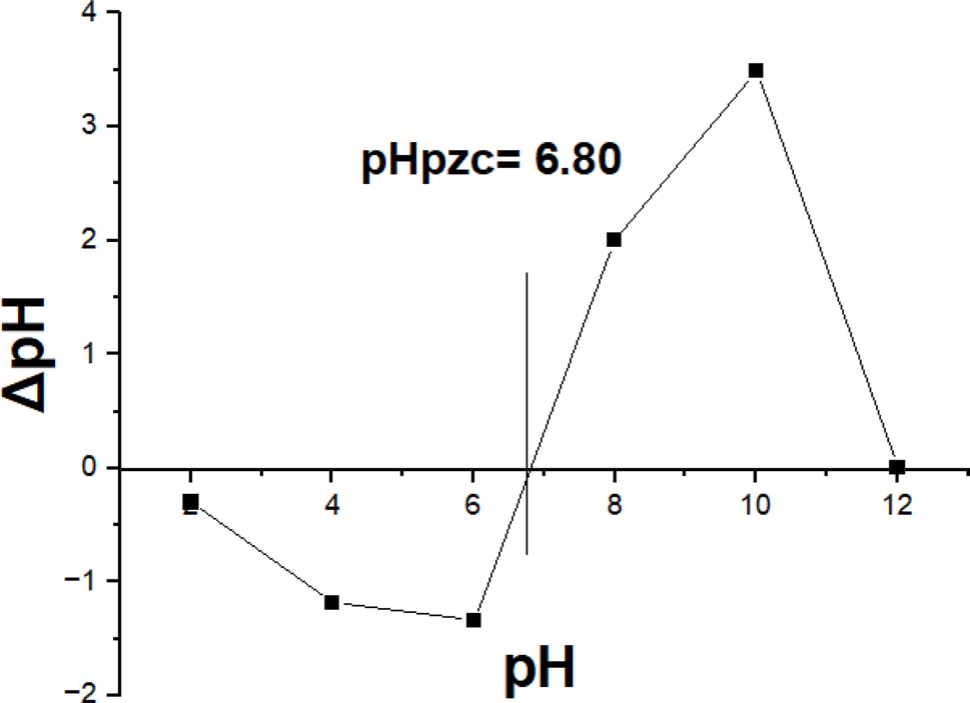
Point of zero fee for MNCPEI adsorbent

The pH of the solution plays a vital role in the adsorption process, significantly affecting adsorption-desorption behavior by altering the surface charge characteristics of the adsorbent (Lin et al. 2024). The effect of pH ranging from 2 to 11 on both dye removal efficiency and adsorption capacity of magnetic nanocellulose polyethyleneimine was examined using 0.1 g of adsorbent and an initial dye concentration of 0.1 g/L, at 27 °C for a contact time of 120 min. Fig. S2 (e) illustrates that the proportion of dye removal escalated from 94.72% at pH 2 to 99.57% at pH 7. The enhancement is due to the alteration in the adsorbent’s surface charge and degree of dissociation of the adsorbate. The findings demonstrate that pH significantly influences adsorption, as increased electrostatic attraction occurs in acidic solutions due to the anionic SO₄^-2^ centres in the RB5 structure, resulting in enhanced adsorption effectiveness at lower pH levels (Elsayed et al. 2022). At higher pH values (8 to 9), both the RB5 removal efficiency and adsorption capacity declined, attributed to decreased protonation of amine functionalities and a rise in hydroxide ions (OH⁻) ion concentration. A likely cause of this decline is the competition at the adsorption sites between OH⁻ ions and dye anions, which disrupts their interaction and decreases total dye uptake (Desai and Kannan 2024). As shown in Fig. 5, the pHₚzc of the adsorbent was measured to clarify its adsorption behavior under varying pH levels. Prior research indicates that when the pH of the solution drops below the pHₚzc, the adsorbent exhibits a net positive surface charge, promoting attraction toward negatively charged dye ions. Conversely, at pH values higher than the pHₚzc, the adsorbent surface becomes negatively charged, resulting in electrostatic repulsion with anionic dyes (Al-Maliky et al. 2021; Tcheka et al. 2024).

While this study was conducted under controlled conditions, it’s important to note that real wastewater often contains salts or surfactants that could affect adsorption. Salts may reduce electrostatic attraction by screening surface charges, while surfactants might compete with dye molecules for active sites or change the surface behaviour of the adsorbent. Despite not exploring this, we consider it relevant and propose it for further research.

### Adsorption modeling and kinetic evaluation

#### Kinetic evaluation

**Fig. 6:**
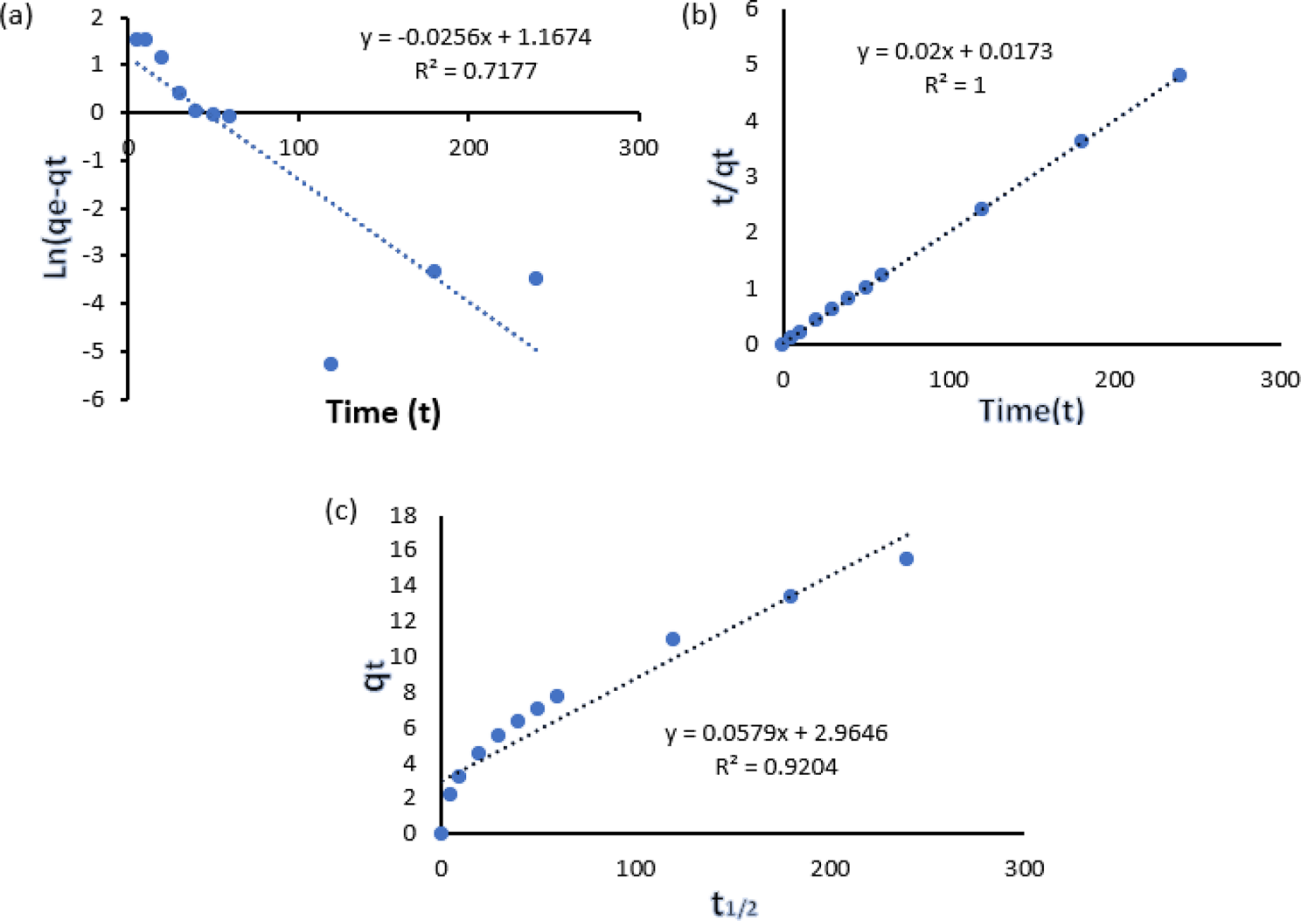
Linear plots showing the (**a**) pseudo-first-order, (**b**) pseudo-second-order, and (**c**) intraparticle diffusion models for RB5 adsorption

Kinetic analysis plays a critical role in clarifying the adsorption pathway and determining how quickly it occurs, aiding in the evaluation of material performance. In this research, the kinetic parameters were determined by investigating the effect of contact time on the amount of Reactive Black 5 (RB5) dye adsorbed by the MNCPEI adsorbent, as presented in Fig. 6(a) and 6(b). Assessment of adsorption kinetics involved fitting the data to both pseudo-first-order and pseudo-second-order models. The following equations represent the models used to describe the rate and underlying mechanism of the adsorption process (Revellame et al. 2020).

4 $${\rm{Pseudo}}\,{\rm{first}}\,{\rm{order:In}}\left({{{\rm{q}}_{\rm{e}}}{\rm{ - }}{{\rm{q}}_{\rm{t}}}} \right){\rm{ = Lng - }}{{\rm{K}}_{\rm{1}}}{\rm{t}}$$

5 $${\rm{Pseudo}}\,{\rm{sconed}}\,{\rm{order:}}{t \over {qt}} = {1 \over {({k_2} \times q_e^2)}} + {t \over {{q_e}}}$$

6 $${\rm{The }}\,{\rm{intraparticle }}\,{\rm{diffusion:}}{{\rm{q}}_{\rm{t}}}{\rm{ = }}{{\rm{k}}_{\rm{i}}}{{\rm{t}}^{{\rm{1/2}}}}$$

Intraparticle diffusion was assessed using the framework proposed by Sajab et al. (2013). In this analysis, qₜ (mg g⁻¹) refers to the dye uptake at a given at a given time t, while qₑ (mg g⁻¹) denotes the adsorption capacity once equilibrium is achieved. The rate constants for the pseudo-first-order and pseudo-second-order kinetic models are expressed as k₁ (min⁻¹) and k₂ (g mg⁻¹ min⁻¹), respectively. Additionally, the intraparticle diffusion constant, k_i_, was determined from the slope of the linear plot of qₜ versus t^1/2^, as illustrated in Fig. 6(c). This model provides further insight into the diffusion mechanism within the pores of the adsorbent during the dye adsorption process (Fig. 6c).

The kinetic plots revealed that the pseudo-second-order model provided a stronger linear fit to the experimental data, with an R² value reaching 1. Additional evidence was provided by the estimated qe values for the pseudo-second-order, which closely aligned with the experimental qe values, suggesting that the adsorption process of RB5 on the MNCPEI surface adhered to the pseudo-second-order model. Figure 6 (a) and (b) display the linear plots of log (q_e_ -q_t_) vs. t for the pseudo-first order and t/q versus t for the pseudo-second order, respectively. Table 3 outlines the kinetic parameters, including rate constants, R² values, and equilibrium adsorption capacities (qeq_eqe​). The pseudo-second-order model provided a better fit for RB5 adsorption onto MNCPEI, as evidenced by its perfect R² value of 1, in contrast to 0.71 for the pseudo-first-order model. As a result, RB5 adsorption onto the MNCPEI is better characterized by the pseudo second-order model. The legitimacy of the prediction is enhanced by the fact that it nearly matches the experimental qe value. The data indicate that chemisorption, driven by electrostatic attraction, predominates over physisorption in the RB5 adsorption process (Zhang et al. 2022a, b).

**Table 3:** Kinetic parameters of RB5 adsorption onto MNCPEI

Kinetic Model	Value RB5
Pseudo-first-order	
q_e_(mg/g)	49.91
K_1_ (min^− 1^)	0.2114304
R^2^	0.717
**pseudo-second-order**	
qe(mg/g)	49.91
K^2^ (g/(mg^− 1^ min^− 1^))	0.0191905
R^2^	1
**intraparticle diffusion**	
q_e_(mg/g)	49.91
R^2^	0.92

The rate-limiting step is determined by the intraparticle diffusion model, which specifies the adsorption mechanism. According to(Donia et al. 2014) when the regression of versus is a straight line passing through the origin, intraparticle diffusion is the rate-limiting step. The existence of two linear segments in Fig. 6(c) indicates that the adsorption process took place in two separate phases. Surface or film diffusion was the first step, followed by adsorption. While intraparticle diffusion was not the rate-limiting element, the RB5 adsorption process was influenced by both intraparticle and film diffusion (Hasani et al. 2022). Dye molecules interacted with functional groups until every available active site was filled. At this stage, there is a substantial adsorption rate because the abundance of functional groups on MNCPEI makes surface adsorption an attractive option for RB5. As the dye molecules diffuse intraparticle through the pores of the adsorbent, the second linear plot shows a gradual adsorption phase. The RB5 dye molecules start diffusing into the pores of MNCPEI once all the surface-active sites are filled. The limited pore capacity of MNCPEI is believed to be responsible for the decreased adsorption rate currently. This suggests that boundary layer diffusion is the primary mechanism for RB5 adsorption onto MNCPEI. Furthermore, an activating step is necessary for enhancing the pore structure; without it, intraparticle diffusion is minimal (J. Wang and Guo 2022). A similar result was also observed in the adsorption of Acid Orange 8 (AO8) and Direct Red 23 (DR23) dyes onto graphene oxide (Lach and Okoniewska 2024).

#### Thermodynamic study

Thermodynamic parameters, such as ∆G° (kJ/mol), ∆H° (kJ/mol), and ∆S° (J/Kmol^− 1^), are important indications for determining the practicality of an adsorption process. Based on the values of these parameters (Table 4), it is feasible to predict whether the process would occur spontaneously (Tsamo et al. 2019).

**Table 4:** Summary of thermodynamic parameters

Temperature (K)	ΔG˚ (KJmol^− 1^)	ΔH^˚^ (KJmol^− 1^)	ΔS^˚^(KJmol^− 1^)
300	1.972232553-	8.250405	33.97608
313	-2.30947		
323	-2.7688		
333	-5.65249		
343	-8.72021		

**Fig. 7:**
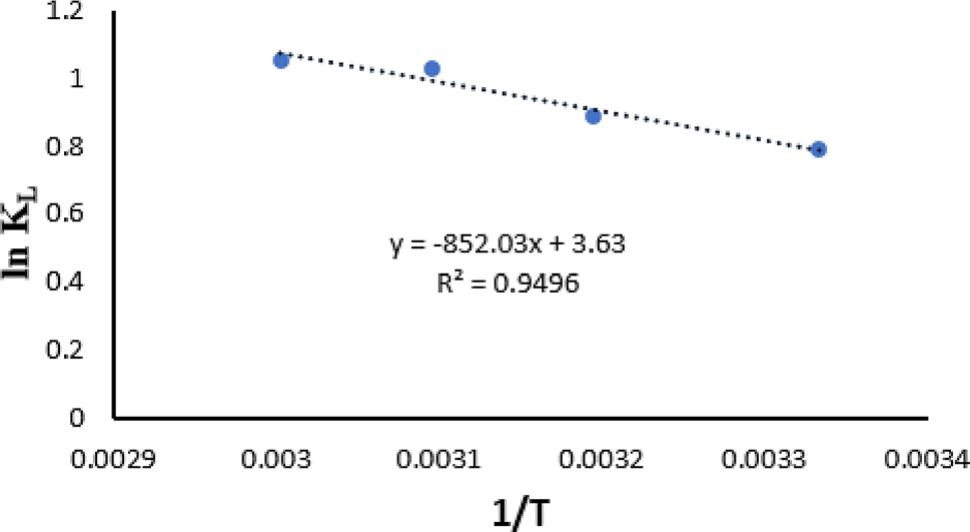
Thermodynamic plot for RB5 adsorption onto MNCPEI

The shift in Gibbs free energy signifies the spontaneity of a chemical reaction, rendering it an essential criterion for assessing whether a process will transpire spontaneously. To compute the Gibbs free energy of the process, it is essential to incorporate both enthalpy and entropy variables (Baghbanbashi et al. 2024; Ebelegi et al. 2020). The objective of the thermodynamic investigation is to identify the characteristics that characterize the adsorption process. The adsorption of RB5 onto MNCPEI was examined at temperatures ranging from 300 K to 343 K. The subsequent equations were employed to ascertain the thermodynamic parameters (Ivanovski et al. 2021; Tran 2022):

7 $${K_L} = {q_e}/{\rm{ }}{C_e}$$

8 $$\Delta {G^ \circ } = {\rm{ }} - RT{\rm{ }}Ln{\rm{ }}{K_L}$$

where KL represents the thermodynamic distribution coefficient of the adsorbent, R denotes the universal gas constant (8.314 J·mol·K^− 1^), and T (K) indicates the reaction temperature. ∆H^o^ and ∆S^o^ values are obtained from the slope and intercept of plot ln K_L_ against 1/T (Ivanovski et al. 2021). Figure 7 depicts the Van’t Hoff plot illustrating the impact of temperature on the adsorption of RB5 on MNCPEI.

The ∆G° values diminish with rising temperature, signifying an enhanced spontaneity and viability for the adsorption of RB5 dye. The Gibbs free energy estimates for the adsorption of RB5 onto MNCPEI are negative across all temperatures. The diminishing ∆G° values with increasing temperature in both dye-anion exchanger systems indicate that elevated temperatures enhance the adsorption process. The negative ∆G° readings indicate that the adsorption process is both advantageous and spontaneous (Syafiqah and Yussof 2018). The negative values of ∆H° further substantiate the exothermic character of the adsorption process, signifying that the adsorption of RB5 dye on MNCPEI is chemical in origin. The positive values of ∆S° indicate a strong affinity of the anion exchangers for the RB5 molecules and suggest an increase in the degree of freedom for the adsorbed species, representing a more advantageous adsorption environment (Lach and Okoniewska 2024; Wen et al. 2019).

It is also important to consider the fate of the MNCPEI adsorbent after repeated use. While the inclusion of magnetic nanoparticles improves recovery and reusability, care must be taken to ensure that these components do not leach into the environment, particularly if the adsorbent is disposed of or used beyond its effective lifespan. Compared to some conventional adsorbents, the use of bamboo-based nanocellulose and PEI offers a more biodegradable and renewable platform. However, further studies are needed to fully evaluate the long-term environmental impact of MNCPEI, especially under field conditions. The adsorption data fit the Langmuir isotherm model very well (R² = 0.994), confirming that RB5 molecules form a monolayer on the MNCPEI surface. This is supported by the pseudo-second-order kinetic model (R² = 1), which indicates that chemisorption is the dominant mechanism.

Further confirmation comes from the surface charge analysis (pH PZC ≈ 6.5), showing that under acidic to neutral pH, the MNCPEI surface carries a positive charge favourable for attracting the negatively charged sulfonic groups of RB5. This explains the enhanced adsorption at lower pH values. The interaction is mainly driven by electrostatic attraction between protonated amine groups on PEI and sulfonate groups in the dye(M. Chen and Hankins 2020). Hydrogen bonding also plays a role, likely occurring between hydroxyl and amine groups on MNCPEI and functional groups (e.g., azo, and sulfonic acid) on RB5. Additionally, π–π interactions between the aromatic rings in RB5 and unsaturated regions in the polymer backbone may further stabilize the adsorption complex. Together, these findings confirm a monolayer, surface-specific adsorption mechanism dominated by chemical interactions (Wang et al. 2024a, b).

### Regeneration

**Fig. 8:**
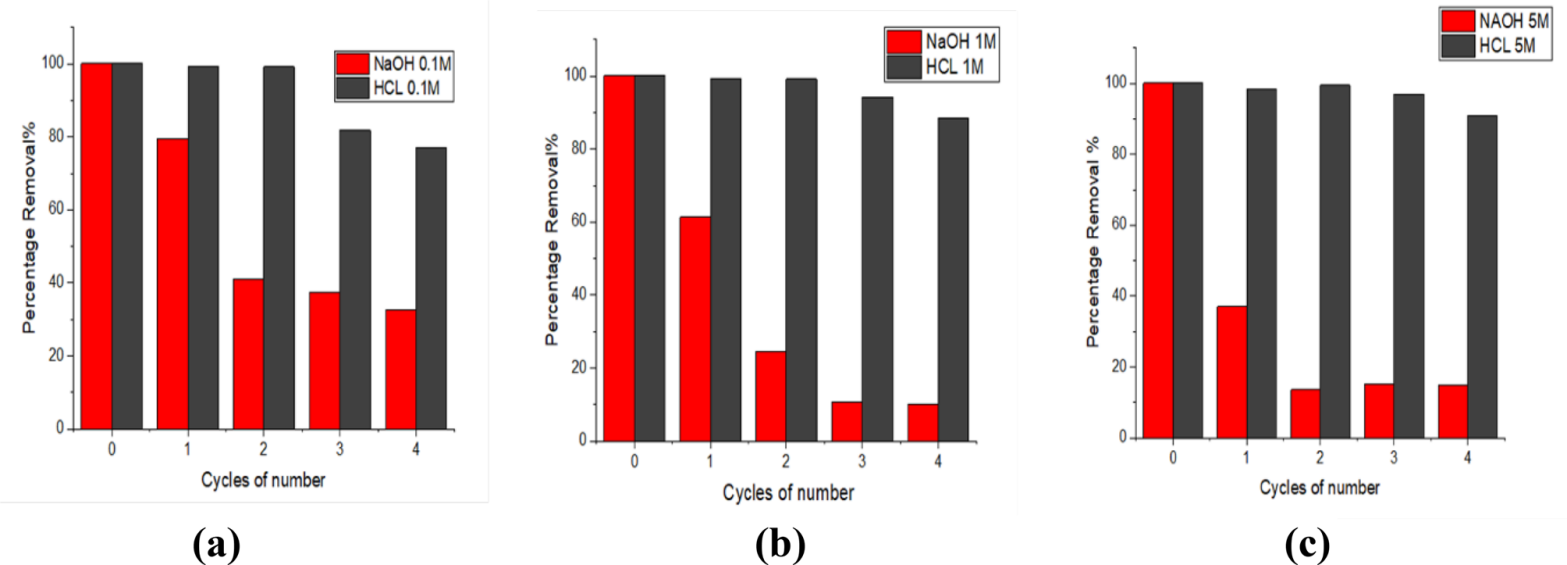
Adsorption efficiencies of the RB5 by acid and base at (0.1,1,5.0 M) (Conditions: initial dye concentration, 0.1 g/L; adsorbent dosage, 0.1 g; sample volume, 50 ml; solution pH, 7; contact time, 120 min; temperature, 27 ℃)

The regeneration of adsorbents is a critical determinant of the efficiency and cost-effectiveness of an adsorption process. The regeneration of utilized adsorbents and their subsequent reapplication in adsorption systems are significant from environmental and economic viewpoints, as they diminish waste and the necessity for new materials, while concurrently reducing the operational expenses of the adsorption process (Renu and Sithole 2024). To assess the reusability of MNCPEI in the study, NaOH (0.1, 1.0, and 5.0 M) and HCl were used as eluents for the regeneration process. The results (Fig. 8(a, b,c) indicate that while NaOH solutions show some ability to desorb RB5, their efficiency is lower than that of HCl solutions at all concentrations (0.1, 1.0, and 5.0 mol/L). As shown in Fig. 8 (a, b, c), the RB5 adsorption peaked at 99% in the initial cycle before gradually declining in following cycles. Possible explanations for the marginal drop in RB5 dye removal efficiency include adsorbent loss during regeneration and the irreversible dye binding to the adsorbent (El Messaoudi et al. 2024; Shoueir et al. 2021). The deprotonation of the adsorbent surface and specific functional groups was also aided by treatment with a basic solution. It is possible that the adsorbent’s surface generated a hydroxyl group, which reduced the electrostatic interaction between the RB5 dye molecules and the magnetic-PEI-nanocellulose adsorbent. This diminished contact may elucidate the decreased adsorption capacity in subsequent cycles (Zhang et al. 2023).

To further understand the long-term applicability of the MNCPEI adsorbent, we examined its performance over multiple reuse cycles. The results showed a gradual decrease in dye removal efficiency—from 99% in the first cycle to approximately 84% by the fourth cycle. This decline suggests that while the material retains good reusability in the short term, its performance may deteriorate with extended use. Potential reasons for this reduction include partial loss or degradation of surface functional groups (especially the amine groups on PEI), irreversible binding of dye molecules that block active sites, and possible structural weakening due to repeated chemical and thermal exposure during regeneration. Unlike many studies that only report the number of cycles reused, our findings begin to shed light on why and how the material’s efficiency diminishes over time.

While some high-capacity adsorbents, such as MWCNTs/PANI/Fe_3_O_4_ composites, offer superior uptake (~ 796 mg/g), their reliance on microwave-assisted desorption and high synthesis cost limits widespread deployment (Elwakeel et al. 2025). In contrast, MNCPEI achieves > 84% dye removal efficiency after four cycles using simple acid or base regeneration, which is more viable in low-resource settings. The minor efficiency loss observed aligns with similar trends reported for chitosan-clay hybrid beads (Khanday et al. 2025), where active site exhaustion and partial functional group degradation occur over time. Despite this, the ease of magnetic separation, absence of toxic eluents, and material biodegradability support MNCPEI’s feasibility in real-world applications. Future optimization of surface chemistry and incorporation of reinforcing agents may further extend its operational lifespan.

### Comparative study

The performance of the MNCPEI adsorbents for RB5 removal was critically compared with various reported adsorbents as presented in Table S1, focusing on maximum adsorption capacity, best adsorption conditions, and adsorption mechanism. It can be observed that raw nanocellulose exhibited low adsorption capacity of 18.5 mg/g, which is expected due to its limited surface functionality and weak interaction with anionic dye molecules of Congo red (Villabona-Ortíz et al. 2022). In contrast, MNCPEI demonstrated significantly higher adsorption capacity of 68.58 mg/g, attributed to the increased density of amine groups that facilitate electrostatic interaction with negatively charged dye species. Notably, the direct modification of sugarcane bagasse fibers with PEI, without prior nanocellulose extraction, resulted in a relatively low adsorption capacity of 25 mg/g for RB5 (Mohamed et al. 2022). This can be attributed to the fact that nanocellulose provides a high surface area, rich hydroxyl functionality, and mechanical stability for anchoring PEI, which Supplies abundant amine groups that enhance electrostatic interactions and hydrogen bonding with the sulfonic groups of RB5. Although commercial AC showed a higher adsorption capacity for the removal of RB5 (198 mg/g), MNCPEI offers significant advantages in terms of low energy consumption during synthesis process of adsorbent(Ip et al. 2010).

Although this study centers on the development of an abiotic magnetic nanocellulose-PEI adsorbent for dye removal, it is important to acknowledge that several prior studies have explored biological and enzymatic approaches for the biodecolorization of Reactive Black 5 (RB5). For instance, (Al-Tohamy et al. 2022b) employed ligninolytic fungi under anaerobic conditions to degrade RB5. The study highlights the potential of biotic systems to disrupt the molecular structure of azo dyes. However, they also underscore important limitations, including longer treatment times, dependency on microbial viability, and sensitivity to environmental fluctuations such as oxygen levels, temperature, and nutrient availability. Furthermore, the generation of secondary metabolites during biodegradation remains a challenge in terms of ecotoxicological safety and effluent quality.

Unlike biological methods, this study presents an abiotic, magnetically responsive MNCPEI adsorbent that removes over 99% of RB5 within 2 h under ambient conditions without needing microbes or post-treatment. It remains highly reusable and environmentally safe, offering greater reliability, ease of scaling, and faster deployment. These advantages make it more practical for industrial wastewater treatment compared to slower, less stable biotic approaches.

## Conclusion

This study successfully developed a magnetic polyethyleneimine-functionalized bamboo nanocellulose (MNCPEI) adsorbent using a glutaraldehyde (GTA) crosslinking technique. The synthesis process was optimized by varying key parameters, with the best performance observed at a nanocellulose-to-PEI ratio of 1:2, a 4-hour impregnation time, 30-minute crosslinking, 1% v/v GTA concentration, and 0.3 g of magnetic nanoparticle (MNP) loading. FTIR analysis confirmed the successful incorporation of PEI and MNPs onto the nanocellulose matrix, while maintaining critical functional groups for dye interaction.

The adsorption of Reactive Black 5 (RB5) was governed predominantly by chemisorption, as demonstrated by kinetic and isotherm models. MNCPEI exhibited high removal efficiency under ambient conditions, supported by pseudo-second-order kinetics and Langmuir monolayer fitting. Magnetic characterization showed that the adsorbent could be recovered within seconds using an external magnetic field, allowing for rapid separation and reuse. Even after four adsorption-desorption cycles, the material retained sufficient structural integrity and magnetic responsiveness for continued application.

Although RB5 is structurally complex and typically resistant to degradation, this study clarified that its removal mechanism under our conditions was based on adsorption rather than chemical breakdown. The functional groups on MNCPEI facilitated strong interactions with RB5 but did not alter the dye’s molecular structure. We recommend future studies to explore potential degradation pathways using advanced techniques such as UV–vis spectroscopy and mass spectrometry to assess any transformation of dye molecules during treatment.

To further contextualize the contribution of this study, a comparative assessment with over a dozen adsorbents reported in the past five years highlights the competitive edge of MNCPEI. While some materials may show slightly higher adsorption capacities, they often rely on narrow pH ranges, complex regeneration procedures, or high-energy synthesis. In contrast, MNCPEI combines high efficiency, operational simplicity, magnetic recoverability, and broad pH tolerance—achieved through a renewable, low-cost, and scalable fabrication process. These integrated advantages underscore the practical value and novelty of this work.

Finally, the findings reinforce the potential of surface-functionalized nanocellulose as an efficient and environmentally responsible solution for dye removal. The straightforward synthesis method and consistent performance across multiple regeneration cycles suggest promising applications for sustainable wastewater treatment. Although post-regeneration structural characterization was beyond the scope of this study, we recommend such analyses in future work to gain deeper insight into adsorbent longevity and mechanisms of performance decline.

## Supplementary Information

Below is the link to the electronic supplementary material.

Supplementary Material 1

## Data Availability

This study article includes all essential data and resources.
