# Supplementary material for: Development and characterization of magnetic polyethyleneimine-bamboo nanocellulose adsorbents for enhanced performance of dye pollutant removal
Source: Bioresour Bioprocess. 2025 Aug 8;12(1):86. doi: 10.1186/s40643-025-00928-y (PMC12334394; doi:10.1186/s40643-025-00928-y)
Supplement: Supplementary file 1 — Supplementary Material 1 [file 40643_2025_928_MOESM1_ESM.docx]

**SUPPPLEMENTARY FILES**

Table S1: List of relevant studies in the literature

| Material | Key Features | Optimal Conditions | Performance | Mechanism & Observations | Ref |
| --- | --- | --- | --- | --- | --- |
| PEI@Zein microparticles | Secondary bond interface assembly; hydrophobic and electrostatic interactions | pH 4–10 | Rapid adsorption (2 min), capacity 631 mg/g | Electrostatic and hydrogen bonding enable efficient RB5 adsorption and easy desorption with 0.01 M NaOH | (Zhuang et al., 2023) |
| Cel/α-Fe_2_O_3_-ZnO nanocomposite | Hydrothermally immobilized on rice husk cellulose; mesoporous structure | pH 2 | Max capacity 99.3 mg/g; reduced multiple pollutants | Adsorption follows Langmuir and second-order kinetics; effective for reactive black 5 and other pollutants | (Aboelfetoh et al., 2023) |
| PbO_2_–g-C_3_N_4_ composite electrode | Synthesized with surfactant templates; durable steel-based electrode | pH 2, 0.15 M Na_2_SO_4_, 60 min | Effective RB5 degradation; stable electrode surface | Electrochemical degradation optimized with surfactants; reusability demonstrated | (Marković et al., 2023) |
| Chitosan-clay hybrid beads (CCHB) | Cross-linked; stable beads with high pore volume | 30–50 °C | Adsorption capacity up to 227 mg/g; negligible decrease after 5 cycles | Adsorption follows Langmuir and pseudo-second order; endothermic and spontaneous | (Khanday et al., 2025) |
| DAC/PEI coated PVDF membrane | Superhydrophilic and superoleophobic membrane | Ambient, tested on oil/water emulsions | >90% dye rejection (RB5 92.3%); flux 350 L/m^2^h | Membrane combines low oil adhesion with dye rejection; effective for complex wastewater | (Liu et al., 2024) |
| Electrocoagulation with aluminum electrodes | Effective for multiple dyes including RB5 | Current density 0.83 mA/cm^2^ | 92.55% RB5 removal; energy efficient | Kinetics fit pseudo-first order; removal varies with dye type | (Omwene et al., 2023) |
| Hybrid constructed wetlands (HCW) | Anaerobic-aerobic treatment with plants enhancing microbial activity | Continuous-continuous feeding, intermittent aeration | 91% colour removal; 99% BOD₅ removal | Plants boost microbial density and pollutant removal; steady feeding reduces toxicity | (Benny & Chakraborty, 2025) |
| Maghemite-coated powdered activated carbon (MAC) + activated sludge (AS) | Magnetic properties for separation; sequential adsorption and biodegradation | pH 7 | >99% RB5 removal; 98.55% COD removal | Combination exploits adsorption and biodegradation advantages; magnetic separation enhances process | (Balci et al., 2023) |
| TiO_2_ nanoparticles on carbon media | Mixed anatase-rutile phases; high surface area | Carbonized at 900 °C | Adsorption of 430 ppm dye in 1 h; photocatalytic efficiency at 700 °C | Mixed phase reduces charge recombination; photocatalysis and adsorption combined | (Limsakul et al., 2024) |
| g-C_3_N_4_/Bi_2_S_3_ carbon-based semiconductor | Hydrothermal synthesis; photocatalytic under UVA | UVA irradiation, 120 min | 97.5% RB5 degradation | Efficient charge separation and radical generation; antibacterial effects | (Rajendran et al., 2024) |
| nZVI@PES membrane with PMS activator | Nanoparticles on membrane; advanced oxidation with reactive oxygen species | pH 3, catalyst 5 mg/cm^2^, PMS 300 mg/L | 96.8% RB5 decolorization in 30 min | Quenching confirms multiple reactive oxygen species; reusable membrane | (Topaloğlu & Kahraman, 2023) |
| UV-C activated persulfate and percarbonate | Life cycle assessment for environmental impact | UV-C activation; RB5 wastewater | 100% RB5 removal | Treatment choice guided by minimal environmental impact | (Dogan et al., 2023) |
| MWCNTs/PANI/Fe_3_O_4_ nanocomposite | High adsorption capacity; microwave regeneration | pH 3 (RB5), pH 10 (benzene) | 796 mg/g RB5 capacity; 4 sorption/desorption cycles | Chemisorption rate-limiting; microwave-assisted desorption confirmed renewability | (Elwakeel et al., 2025) |
| Chitin magnetite nanoparticles (ChM) | Ultrasound-assisted synthesis; pH-dependent adsorption | pH 4 modified, pH 8 unmodified | Effective adsorption; size and electrostatics influence process | Sustainable, inexpensive; potential alternative adsorbent | (Indira et al., 2023) |
| Ternary metal composites (TMCs) | Variable aluminum salts tailor adsorption selectivity | Various Al salt precursors | Selective anion adsorption; high arsenate removal | Physisorption dominates for dyes; chemisorption for arsenate and fluoride | (Steiger & Wilson, 2023) |


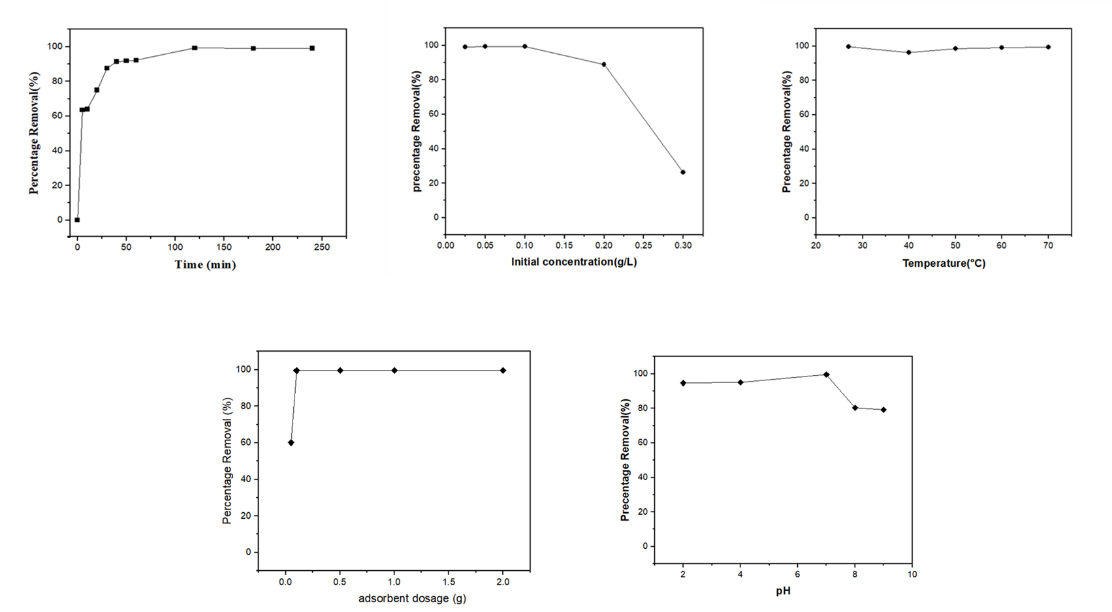

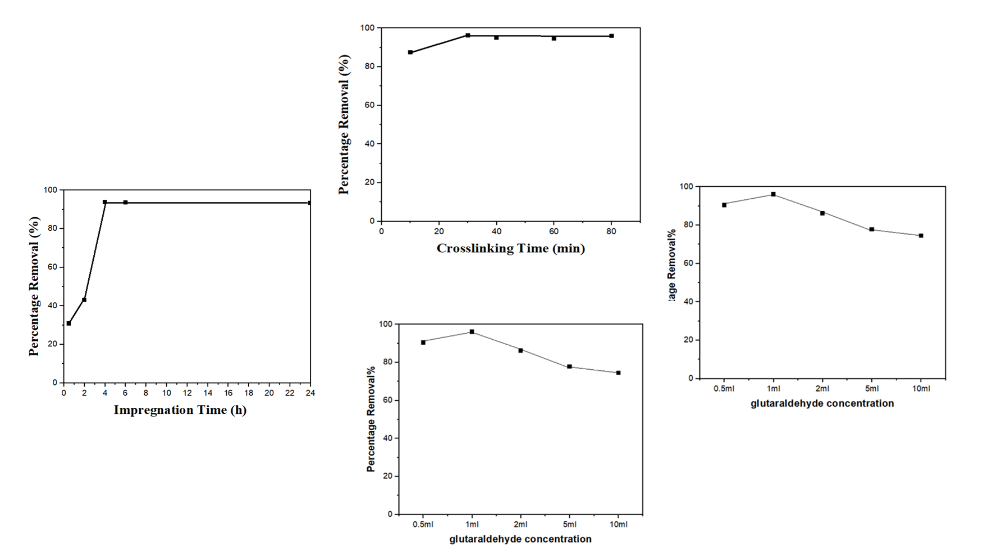


Figures S1& S2 (a, b, c, d) of Effect of Adsorption Parameters & Effect of Adsorbent Preparation Parameters

**References**

Aboelfetoh, E. F., Elabedien, M. E. Z., & Ebeid, E.-Z. M. (2023). In situ anchoring of iron and zinc oxides nanoparticles onto rice husk cellulose for efficient wastewater remediation. *International Journal of Biological Macromolecules*, *233*, 123562.

Balci, B., Toprak, S., Erkurt, F. E., Zaimoglu, Z., Basibuyuk, M., Budak, F., Yesiltas, H. K., & Turan, E. S. (2023). Sequential Fe2O3-powdered activated carbon/activated sludge process for the removal of Reactive Black 5 and chemical oxygen demand from simulated textile wastewater. *International Journal of Environmental Research*, *17*(1), 11.

Benny, C. K., & Chakraborty, S. (2025). Effect of Typha angustifolia, feeding modes and intermittent aeration on the performance of hybrid constructed wetland systems treating Reactive Black 5 diazo dye wastewater. *Environmental Science and Pollution Research*, *32*(3), 1581–1597.

Dogan, K., Atilgan Turkmen, B., Germirli Babuna, F., Koba Ucun, O., & Arslan Alaton, I. (2023). Merging treatability results and sustainability assessment: a segregated textile dyehouse effluent. *International Journal of Environmental Science and Technology*, *20*(10), 11165–11176.

Elwakeel, K. Z., Elgarahy, A. M., Zoromba, M. S., Al-Bogami, A. S., Akhdhar, A., & Mashaal, N. M. (2025). Optimizing Magnetic Carbon Nanotube/Polyaniline Nanocomposite for Sequestering Harmful Organic Contaminants. *Water, Air, & Soil Pollution*, *236*(4), 205.

Indira, P., Ho, T.-T., Ahalya, N., Sathish, T., Saravanan, R., Rajasimman, M., & Sudhakar, T. (2023). Magnetic porous Ag2O/Chitin nanostructure adsorbent for eco-friendly effective disposing azo dyes. *Environmental Research*, *218*, 114824.

Khanday, W. A., Soon, K. A., Ahmed, M. J., & Hummadi, E. H. (2025). Cross-linked chitosan/pencil clay hybrid beads for the adsorption of reactive black 5. *International Journal of Biological Macromolecules*, *285*, 138301.

Limsakul, S., Thanachayanont, C., Siyasukh, A., Jaideekard, M., Yimklan, S., Kijjanapanich, P., & Chimupala, Y. (2024). High efficiency azo dye removal via a combination of adsorption and photocatalytic processes using heterojunction Titanium dioxide nanoparticles on hierarchical porous carbon. *Environmental Research*, *260*, 119627.

Liu, J., Huang, Y., Zhang, G., Wang, Q., Shen, S., Liu, D., Hong, Y., & Wyman, I. (2024). Dialdehyde cellulose (DAC) and polyethyleneimine (PEI) coated polyvinylidene fluoride (PVDF) membrane for simultaneously removing emulsified oils and anionic dyes. *Journal of Hazardous Materials*, *471*, 134341.

Marković, A., Savić, S., Kukuruzar, A., Konya, Z., Manojlović, D., Ognjanović, M., & Stanković, D. M. (2023). Differently prepared PbO2/graphitic carbon nitride composites for efficient electrochemical removal of Reactive Black 5 dye. *Catalysts*, *13*(2), 328.

Omwene, P. I., Can, O. T., Öz, U. M., & Keyikoğlu, R. (2023). Investigating the removal efficiency of different textile dye classes from wastewater by electrocoagulation using aluminum electrodes. *International Journal of Environmental Science and Technology*, *20*(12), 13009–13020.

Rajendran, R., Rojviroon, O., Arumugam, P., Natchimuthu, K., Vasudevan, V., Kannupaiyan, J., Muangmora, R., Phouheuanghong, P., & Rojviroon, T. (2024). Design and fabrication of g-C3N4/Bi2S3 heterojunction photocatalysts for efficient organic pollutant degradation and antibacterial activity. *Journal of Alloys and Compounds*, *976*, 173116.

Steiger, B. G. K., & Wilson, L. D. (2023). Sustainable hybrid biocomposite adsorbents for anion-selective or concerted removal of ionic pollutants: Organic dyes to arsenate. *Journal of Cleaner Production*, *422*, 138616.

Topaloğlu, A. K., & Kahraman, B. F. (2023). Textile dye removal in wastewater by peroxymonosulfate (PMS) activation on a zero-valent iron nanoparticle–modified ultrafiltration catalytic membrane (nZVI@ PES). *Environmental Science and Pollution Research*, *30*(41), 94779–94789.

Zhuang, Z., Cheng, X., Cao, L., He, G., Zhou, J., & Wei, Y. (2023). Secondary bond interface assembly of polyethyleneimine on zein microparticles for rapid adsorption of Reactive Black 5. *Colloids and Surfaces B: Biointerfaces*, *225*, 113247.
